# Help-seeking for Intimate Partner Violence and Abuse: Experiences of Civilian Partners of UK Military Personnel

**DOI:** 10.1007/s10896-022-00382-w

**Published:** 2022-04-05

**Authors:** Filipa Alves-Costa, Rebecca Lane, Rachael Gribble, Anna Taylor, Nicola T Fear, Deirdre MacManus

**Affiliations:** 1 Department of Forensic and Neurodevelopmental Sciences, Institute of Psychiatry, Psychology and Neuroscience, King’s College London, 16 De Crespigny Park, Camberwell, London, SE5 8AB UK; 2 King‘s Centre for Military Health Research, King‘s College London, Weston Education Centre, 10 Cutcombe Road, London, SE5 9RJ UK; 3 Research Department of Clinical, Educational and Health Psychology, University College London, 1-19 Torrington Place, London, WC1E 7HB UK

**Keywords:** Intimate Partner Violence and Abuse (IPVA), UK Military, Civilian spouses, Help-seeking

## Abstract

There is evidence that Intimate Partner Violence and Abuse (IPVA) is more prevalent among military populations compared with civilian populations. However, there has been limited research into the help-seeking experiences of civilian victim-survivors who have experienced IPVA within relationships with military personnel. This qualitative study aimed to explore the experiences of, and barriers to, help-seeking for IPVA victimisation among civilian partners of military personnel in order to identify strategies to improve the management of IPVA both within the military and civilian sectors. The study adopted a descriptive cross-sectional study design and used qualitative research methods. One-to-one telephone interviews were conducted with civilian victim-survivors (n = 25) between January and August 2018. Interview transcripts were analysed using thematic analysis. Three superordinate themes were derived: (1) Drivers to help-seeking; (2) Barriers to help-seeking; and (3) Experiences of services. The findings indicate difficulties in help-seeking for IPVA among civilian partners of military personnel due to stigma, fear, dependency, poor understanding of IPVA, lack of appropriate and timely support, and a perceived lack of victim support. Difficulties in help-seeking were perceived by participants to be amplified by military culture, public perceptions of the military, military protection of personnel and the lack of coordination between civilian and military judicial services. This study reinforces the need for a military specific Domestic Abuse strategy, identifies vulnerable groups and highlights a need for both increased awareness and understanding of IPVA within civilian and military services in order to provide adequate victim protection.

## Introduction

Intimate Partner Violence and Abuse (IPVA), defined as “*any behaviour within an intimate relationship that causes physical, psychological or sexual harm to those in the relationship”* (World Health Organisation, 2012), is of growing concern, particularly due to increased frequency and severity observed during Covid-19-related restrictions in the UK (Campbell, 2020; Usher et al., 2020). There is evidence to suggest that the prevalence of IPVA perpetration may be higher in military populations compared with civilian populations (Kwan et al., 2020; MacManus et al., under review). There are factors related to military service which may increase the likelihood of IPVA occurrence. Some have portrayed military culture as one of machismo and legitimised violence and have described how violence can *spill over* into the home (Bradley, 2007; Gee, 2017; Jones, 2012). Military-related relocations and periods of imposed separation and reintegration, such as those relating to deployment, can place unique stressors on relationships and have been associated with increases in IPVA (Alves-Costa et al., 2021; Dichter et al., 2015; Knobloch & Theiss, 2012). Furthermore, mental health difficulties (some deployment-related), such as Post-Traumatic Stress Disorder (PTSD) and alcohol misuse, which are reportedly greater in military populations (Goodwin et al., 2015; Rhead et al., 2019), have been reported to be associated with IPVA perpetration in military personnel (Alves-Costa et al., 2021; Cancio & Altal, 2019; Kwan et al., 2020; MacManus et al., under review; Trevillion et al., 2015).

Despite these findings, there remains a paucity of research exploring experiences of help-seeking for IPVA among military populations, particularly those of civilian spouses or ex-spouses who are on the margins of both civilian and military communities (Gray, 2015). Experiences of help-seeking for IPVA have been well-documented in civilian populations and multiple barriers have been identified. These barriers can arise at the level of the individual, such as self-blame, stigma, and fear of repercussions (Murray et al., 2018; Overstreet & Quinn, 2013; Rose et al., 2011), and also at service level, including lack of awareness of and trust in services (Huntley et al., 2019), and perceived lack of staff training and skill in identifying and managing IPVA (Ramachandran et al., 2013; Rose et al., 2011; Sprague et al., 2012). Given the additional stressors of military life, it is likely that there are particular complexities to help-seeking for those in abusive relationships with military personnel. The UK Ministry of Defence (MOD) has identified factors which may deter reporting of IPVA and its management, for instance dependence of the spouse or partner on the perpetrator for financial support or a perception that the military will protect the perpetrator and not support survivors (Ministry of Defence, 2018). Limited UK research into help-seeking for IPVA by civilian spouses of military personnel has identified further barriers to include fear of the impact on the career of military personnel (Williamson, 2012; Williamson & Matolcsi, 2019), a perceived lack of confidentiality within military welfare services (Gray, 2015, 2016; Williamson, 2012; Williamson & Matolcsi, 2019), and a perceived ineffectiveness of support available for IPVA within the context of the military (Gray, 2016). US research has identified military protection of personnel, lack of safe spaces and financial dependency as key factors which deter help-seeking among military wives (Kern, 2017) and that foreign nationals experience additional complexities to help-seeking for IPVA compounded by their migrant circumstances and status (Gray, 2016; Williamson & Matolcsi, 2019).

Research to date has been limited by small sample sizes and low rates of self-disclosed IPVA within those samples; a narrow focus on partners of deployed and regular personnel; or inability to extrapolate from the US to the UK context. Qualitative exploration of how military life can impact experiences of help-seeking in civilian victim-survivors of IPVA from relationships with UK military personnel is needed for a more in-depth understanding of civilian partner experiences and to guide the development of support services for this important subgroup of victim-survivors. Increased understanding of how being in an abusive relationship with someone in the military may give rise to different experiences of help-seeking would also significantly benefit upcoming reviews of the MOD Domestic Abuse Strategy (2018). The current study aims to highlight the challenges in help-seeking for IPVA experienced by civilians whilst in relationships with military personnel to improve care provision and support. It was guided by the following questions to explore civilian partners of serving or ex-serving military personnel’s experiences of help-seeking for relationship difficulties and IPVA:

1. What are the facilitators and barriers to civilian partners accessing support for IPVA and related difficulties?
2. Are there military specific factors which affect civilian partners of military personnel’s help-seeking journey?
3. What are civilian partner experiences of receiving support from services for IPVA and related difficulties, and are these different across military and civilian services?

## Method

### Study Design

This research was undertaken as part of a wider mixed-methods study into IPVA in military couples (defined as couples in which one or both partners are serving or has served in the UK military; see Alves-Costa et al., 2021; Lane et al., under review-a, under review-b, under review-c, under review-d; MacManus et al., under review). This descriptive cross-sectional study used qualitative phenomenological research methods and a critical realist approach to explore the help-seeking experiences of civilian victim-survivors of IPVA within intimate relationships with serving personnel and veterans.

### Recruitment

Individuals (all genders) who identified as civilian victim-survivors of IPVA that occurred within relationships (heterosexual and homosexual) with military or ex-military personnel (henceforth referred to as *(ex)partners)* were eligible for inclusion. Exclusion criteria included individuals who identified as victims-survivors of IPVA perpetrated by civilian partners or if they had previously served in the military. Participants were recruited from several national military and civilian welfare support charities, clinical services for serving personnel, veterans and their families, and specific support organisations for victim-survivors of IPVA. Prior to study involvement, participants received study information and a sign-posting booklet containing information on support services, and provided written consent. Participants were offered £25 as compensation for their time.

### Participants

A total of 25 participants were interviewed between January and August 2018. All participants were women in heterosexual relationships and described being the victim of moderate to severe unidirectional IPVA (see Alves-Costa et al., 2021; Lane et al., under review-c). Participant’s mean age was 42.2 years and the majority described themselves as White (23/25) or British (22/25). See Table 1 for participant socio-demographics and military (ex)partners military characteristics.

**Table 1:** Participant demographics and military characteristics of (ex)partner

	*n*
**Age** (years)	
< 35	6
35–49	12
*50+*	7
**Ethnicity**	
*Minority ethnic group*	3
*White*	*22*
**Branch** *	
*Royal Navy/Royal Marines*	6
*Royal Air Force*	2
*Army*	21
**Serving status**	
*Ex-serving (veteran)*	16
*Serving*	11
**Engagement status** *	
*Regular*	27
*Reservist*	4
**Rank**	
*Officer*	3
*Non-Commissioned Officer (NCO)*	14
*Other rank*	8
*Unknown*	2
**Length of service (**years)	
*5 to 14*	11
*15 to 24*	11
*25+*	2
*Not known*	3
Deployment experience**	
*Deployed*	27
*Not deployed*	0

Two participants reported more than one abusive relationship with a military person, with the total sample reporting on 27 abusive relationships with military personnel

^*^These groups are not mutually exclusive. Some military (ex)partners were reported to serve in multiple Service branches and as both regular and reservist military personnel

^**^Deployment experience does not include detail on whether military personnel held combat roles on deployment, although participant narratives would suggest this was common

### Data Collection

Following Patient and Public Involvement (PPI) consultation, a semi-structured interview schedule was developed. The topic guide was comprised of two sections: (i) participant experiences of IPVA and the perceived impacts on themselves and their children; and participant perceptions of the impacts of military life on intimate relationship(s) and IPVA (for analysis of this data see Alves-Costa et al., 2021; Lane et al., under review-c); and (2) participant experiences and attitudes regarding help-seeking for IPVA, as well as participant suggestions on what they found/would have found helpful. In total, 8 open questions were asked to elicit the participant’s narrative regarding perceived facilitators and barriers they experienced to access support for IPVA; military specific factors which affected their help-seeking journey; and usefulness of care provision from civilian and military services and recommendations for policy and practice. Example questions include: *How did you find the process of asking for/seeking help? Do you think being in a relationship with someone in the military had an impact on you seeking help?* One-to-one interviews were conducted over telephone and (lasted between one to two hours. Telephone interviews are deemed appropriate to facilitate engagement by providing a sense of participant anonymity (Mealer & Jones, 2014) and to recruit participants over a broad geographical area. To note that all interviews were scheduled considering participants' availability and all were requested to take part in the interview in a private space where they felt safe and comfortable to talk with the researcher. Interviews were digitally recorded, transcribed verbatim for analysis and anonymised to protect participant identity. Data saturation informed data collection.

A risk management plan was developed due to the potentially distressing nature of the interviews. A sign-posting booklet containing information on support services was given to potential participants. All participants interviewed were offered debriefing and the opportunity to speak with the study medical officer (DM), which was taken up by one participant.

### Ethical Approvals

Ethical Committee approval was granted by the King's College London Psychiatry, Neuroscience and Midwifery Research Ethics Committee (Ref HR-17/18–5356).

### Analysis

Interviews were analysed using Thematic Analysis (Braun & Clarke, 2006). After a process of *familiarisation*, a *coding framework* was developed based on the interview topic guide and simultaneous coding of the first six interview transcripts by two researchers (FAC and AT), simultaneously implementing an inductive and deductive approach. This initial framework was applied to the remaining transcripts and initial themes were generated where meanings in the data were identified and related to each other. The suitability of the coding frame and any discrepancies during the data analysis process were assessed through progressive iterations and discussions within the research team, revisited until the write up was finalised. The reflexive process and input from key stakeholders and PPI guided the researchers in finding and understanding patterns of meaning within the data. This also helped the researchers be both close to and distant from the data. The analysis process was complimented by the principal investigator (DM) and an independent moderator (RL) using iterative categorisation (Neale, 2016) in an effort to verify coding and draw out finer nuances in the data. Data management was supported by QSR NVivo12 software (QSRInternational, 2018).

### Patient and Public Involvement

A PPI event was organised, involving consultation with professionals (military research, IPVA research and services, mental health research and services, members of the Armed Forces) and civilians with personal experience of abuse by their military (ex)partners. Feedback from this group informed the topic guide used to conduct the interviews, refine the framework and validate our findings, which minimised bias and ensured that the final findings presented in this manuscript represent the participants’ experiences.

PPI in research has become increasingly important over the past 10–15 years. Participants, patients and careers are the end users of health services research, and it is good practice to involve them when conducting research, hence considered by the research team. PPI has the potential to improve the relevance and quality of scientific clinical research.

### Reflexivity Statement

It is important to note that all authors are White European, female, have never served in any Armed Forces, and have undertaken postgraduate study. Authors have no current or previous affiliations to the Ministry of Defence or military. It is possible that author characteristics and pre-conceptions of the military and/or of IPVA may have influenced participant responses and affected the way the interviews were conducted, and the analysis was approached. However, the non-military serving status of interviewers was considered likely to reduce barriers to disclosing issues with the military institution and principles of reflective practice were used in team discussions to help identify and understand author perspectives. Furthermore, consultation with senior researchers and practitioners with expertise in military families research and/or IPV throughout the course of the study enabled the team to make procedural decisions, discuss details of data generation and management, enhancing trustworthiness, and supported our reflexivity, minimising the possibility for bias.

## Findings

Three primary themes were identified from the data; drivers of help-seeking; barriers to help-seeking; and experiences of services (Table 2).

**Table 2:** Themes and subthemes derived from thematic analysis

Themes	Subthemes
• Drivers of help-seeking	*i. Experiences of heightened abuse* *ii. Protecting children* *iii. A support network*
• Barriers to help-seeking	*i. Individual-level barriers* *ii. Relationship barriers* *iii. Service-level barriers* *iv. Societal barriers*
• Experiences of services	*i. Military health and welfare services* *ii. Civilian health and welfare services (charity, housing, social and NHS services)* *iii. Police and the Justice system* *iv. Military / civilian divide*

### Theme 1: Drivers of Help-seeking

Theme 1 describes the drivers of help-seeking for IPVA and related problems reported by participants. Three subthemes were identified: *Experiences of heightened abuse; Protecting children*; and *A support network.*

#### Experiences of Heightened Abuse

Many participants described relationship breakdown and help-seeking for IPVA as being triggered by more extreme IPVA or escalations in abuse and violence.

*I waited until he calmed down and he put the knife down, and he had gone into another room to tidy something up or something like that, and I sneaked past him on the stairs and ran out the house. […] So I didn’t tell him, ‘I’m leaving you,’ but, when the police arrived and they arrested him, that was me saying that’s it.* (P3)

*I had to run out of the house because he told me that he was going to kill me. It was the worst attack by far. […] I ran to the guard room, and they phoned the military police and they went into the house, broke down the front door, arrested him.* (P21)

#### Protecting Children

Participants with children shared that having to protect their child/ren was often a motivator for leaving or reporting the abuse:

*He was holding a hammer up above my head and my daughter, who was four at the time, just walked in and asked if she could have a packet of crisps. […] She wasn’t shocked. She wasn’t anything. That is when I thought, ‘I’ve got to leave.’ I never did it for myself.* (P12)

*[(Ex)partner] was so violent, my son got involved in the attack where he hurt my son as well. I called the police and they had to remove him from the property. (*P7*)*

Reporting IPVA when children were involved often resulted in participants being put in contact with external organisations, which was reported as helping them to realise the severity/risk of the experiences faced both by themselves and their children.

*When it was just me and him, and very much just behind closed doors, there weren’t a lot of ripples, if you like, from his behaviour. But, as soon as we have the children, and there were incidents, then the police were called, and then social services notified and army welfare were notified, and sometimes the children’s school was notified.* (P8)

#### A Support Network

When participants described the contexts in which they sought help, some recalled instrumental practical and emotional support from family, friends and colleagues, for instance providing them with a safe space or supporting them with reporting IPVA and accessing services. A minority noted that external agencies helped them recognise their experiences as abuse and directed them to appropriate services.

*The second time, when he assaulted me, I don’t think I knew I was leaving right there and then. Funnily enough I called a friend. I texted her, and she came round, and then she let my family know, and she contacted the police.* (P9)

* [Service name] is a charity, but they were obviously specialised with military. [Relationship counsellor] identified [IPVA] within five or 10 minutes of us talking to her. I remember just sitting there in shock for weeks thinking, ‘What does she mean this is abuse?’ It took me a long time because I think I had been brainwashed. I had been brainwashed into [thinking] this is normal.* (P23)

### Theme 2. Barriers to Help-seeking

Theme 2 outlines barriers to help-seeking as described by participants and was comprised of four subthemes reflecting different levels of participants’ socio-ecological systems: *Individual-level barriers; Relationship barriers; Service-level barriers*; and *Societal barriers*.

#### Individual-level barriers

##### Lack of Understanding of IPVA

A lack of awareness of all forms of IPVA, especially non-physical forms of IPVA, was described throughout participant interviews and resulted in participants not recognising the abuse until more extreme physical or sexual violence occurred. This also related to (ex)partners’ perceptions of IPVA and denial of abuse because it may not have presented as direct physical abuse, e.g. hitting/punching.

*The insults and the put-downs, the coercive control just became part of everyday life. I didn’t even realise it was abuse.* (P2)

*I was at the top of the stairs, and he got me by my throat […] He just suspended me back and threatened to drop me. The threat again, not the hitting, because that would be wrong.* (P20)

Most participants described normalising relationship difficulties and IPVA behaviours, pretending the abuse was not happening or blaming themselves. Participants shared that experiences of psychological abuse fed into self-blame narratives, identifying the participant as “*the problem*”. This was facilitated by a perceived culture of normalisation and minimisation of violence in the military and resulted in participants failing or delaying to seek help for IPVA.

*I felt like it was probably just me, and that probably I was overreacting and that everybody probably had exactly the same experience, but they just coped with it better than I did*. (P8)

*When you are in it, you can’t fully see what is going on. […] he had convinced me that everyone else was lying or I had misunderstood things or he started calling me a silly sausage or, ‘You know it was like that,’ […] it is called gas-lighting.* (P9)

##### Fear

For many participants, fear of reporting the abuse and leaving the relationship was identified as a key barrier to seeking help. Participants described that fear of retribution and punishment, borne of the threats received or direct interference in their help-seeking efforts, often prevented them from leaving and seeking help for their relationships.

*He used to threaten me with what he would do to me if I left him as well: ‘I could make it look like suicide,’ and things like this.* (P10)

*I dialled The Samaritans once, and he [(ex)partner] cut the telephone cord. […] That was the last time I ever asked for help.* (P4)

Most participants who had not sought help while in the relationship described fear of the impact that reporting IPVA could have on their (ex)partners’ career. They voiced strong preferences for preserving their (ex)partners’ careers and status, even to their own detriment. Participants also expressed fear as other members of the military told them reporting their (ex)partner could make the abuse worse.

*They [military welfare] explained to me that, if he was to do it again, they would have to phone the police and they wouldn’t really have any other choice. So that stopped me going in the next few times, because I was too worried. I didn’t want to phone the police. What I wanted was some help for him.* (P3)

*His immediate superior […] really wasn’t interested. He just told me that, if I made a fuss, he would be downgraded and it would affect his career and it would make him probably more angry. […] He just told me to keep quiet*. (P20)

#### Relationship barriers

##### Isolation and Dependency

Although some participants described receiving support from informal sources in accessing IPVA services, many described how military-related relocations, control by their (ex)partners, or a need to hide IPVA resulted in increased social isolation, hindering opportunities for disclosure.

*Because I wasn’t allowed to talk to people and wasn’t allowed to see anybody, I didn’t have anywhere to turn to or anyone to go to. It was hard to get out of the relationship. (P*19*)*

*Nobody comes to the house. If I go anywhere, he has to be with me. […] I used to have to lie and say I was going to hospital, because I knew he wouldn’t come with me because it is too difficult on the buses [due to physical disability].* (P25)

Participants also identified social, emotional and financial dependency on their (ex)partners as a barrier to seeking help. This was described to be amplified by military-related relocations, especially overseas, which could increase participant isolation from family and friends and interrupt independent career development.

*[Relocating] had an impact on my career. I think it isolated me. It took me away from my friends and family, and I found it really difficult to make new friends.* (P15)

Financial dependency was associated with participants having reduced options if they left their relationship, for instance as a result of not having housing or challenges in obtaining legal support.

*Because, for me, he had a job, he could get a lawyer, probably help from the army if he needed it, he had a house. I would literally be homeless, and they are not going to let me have my kids, when he has got a full-time job and he has got a house to keep them safe. (P*11*)*

*[The police] won’t do anything until the non-molestation order is in place, but, because I can’t afford a solicitor, that kept getting delayed because I didn’t really know what I was doing with the paperwork. I have had to write my own statements without any help. (*P24)

Isolation and dependency was also identified as a particular difficulty by Foreign and Commonwealth participants, who were particularly likely to be socially isolated from informal sources of support and additionally relied on their (ex)partners for the right to remain in the UK.

*[In home country] when I got beaten I would just go to my parents’, but, […] when I came to Germany, I was really isolated because my friends had their husbands but they never got a […] beating. So I just stayed there with no one to talk to.* (P22)

*The main reason I was staying with him was because of my papers to stay in the UK.* (P22)

In some cases, the fact that housing provided by the military was in the (ex)partner’s name created additional complications for participants’ protection and increased dependency on (ex)partners. This was perceived by participants to limit the ways in which the military could intervene to support and protect them.

*Because his name was on [the house], even though the police had said he couldn’t be near us or the kids, the army couldn’t do anything when he broke in, because, technically, he hadn’t. (P*11*)*

##### Being a ‘Good’ Military Wife

Some participants explained how, in spite of the severity of the abuse they endured, they stayed in the relationship because of love for their (ex)partners, hopes their (ex)partners behaviours would change, or guilt at the thought of breaking up their family unit if they were to leave. For these participants, the discussion largely centred on self-blame for the violence they faced.

*I lived on the hope that he would change, I guess. If I was just a better wife, it would stop.* (P23)

*There was fear of my children growing up without a father because of the whole stigma that children should have both parents*. (P23)

Reflecting the culture of loyalty and spousal support in the military depicted by participants, many described attempts to obtain support for their (ex)partner’s mental health, often before or while seeking help for themselves, delaying or jeopardising their access to support.

*When I left once when my daughter was one, I went into a refuge and I was actually rehomed, but I went back to him after four weeks. But, at the time, I was trying to get him help for his PTSD and his drinking. (P*12*)*

#### Service-related barriers

##### Lack of Awareness of Services

Some participants described being unaware of where they could seek help from and avenues to accessing support, especially Foreign and Commonwealth participants or those on a base away from their local area or posted overseas, which increased their vulnerability and prevented them from leaving.

*There was nothing in Germany. I wouldn’t have even known where to look.* (P10)

*No one knows what is available to them, and knowledge is power. People […] should be able to feel that they are going to be supported outside of the army. They don’t know what benefits are available. They don’t know where they are going to be housed. They don’t know their own rights, and that is what stops most people from leaving*. (P21)

##### Perceptions of Services

Previous negative experiences with services created additional barriers for participants. These revolved around a general mistrust of services and a lack of confidence in their ability to help and safeguard. Particular concerns were raised over increased risk and escalation of IPVA if services were not able to secure conviction or protect participants.

*I was really scared. I wanted to leave. I had tried to leave in the past and it backfired because the welfare officer had gone to him. I didn’t trust anybody.* (P23)

*When I was in the situation [the relationship], my biggest fear was that one of these agencies was going to end up making the situation worse and actually result in me dying.* (P15)

Participants also reported instances of victim-blaming, particularly by Social services or the police, which they viewed as maintaining silence among victims and contributed to service mistrust.

*They [social services] threatened me with removing my children if I wasn’t protecting them from an abuser. [...] they did nothing to have him charged for the abuse.* (P20)

##### Service Access

Some participants described challenges in accessing services, particularly in instances where the severity of their abuse was not recognised or if their (ex)partners were not willing to engage.

[Welfare] *couldn’t really help me because it wasn’t him that was contacting. Unless they felt that he was in danger or he was putting others in danger, they couldn’t speak to him about it. […] His temper was just verbal. […] because it wasn’t physical and he hadn’t approached them, they couldn’t do anything.* (P1)

*They could only help me up to a certain point. You needed to have that other person buy into whatever was being offered. […] you needed to have both; you needed to have the other person recognise that there was something wrong, and [ex-partner] wouldn’t do that.* (P18)

#### Societal barriers

##### Shame and Stigma

Many barriers to help-seeking for IPVA related to perceived/anticipated stigma. These included embarrassment or shame experienced by participants as a result of their abusive relationship, resulting in non-disclosure. Participants also described experiences of criticism for not having left the relationship, or saw their experiences ‘normalised’ or justified reflecting a misunderstanding and misperception of IPVA.

*You feel embarrassed and you feel like you should have known better and you should have seen the signs […] You feel like you have done something wrong.* (P3)

*You tend to find that there is this assumption, like my friend’s husband again –remember him saying to me, ‘Oh, you’re not easy to live with.’ […] I found that society still wanted to blame* [the victim-survivor] *because it is easier.* (P20)

Some participants attributed IPVA experiences to their (ex)partners’ mental health, for which help-seeking was also perceived to be associated with significant stigma in the military. (Ex)partner mental health difficulties, and correspondingly IPVA, were felt to be perpetuated by a lack of military understanding of and support for mental health and family issues, as well as a toxic culture of machismo which views help-seeking as weakness. Concerns were also raised over the perceived impact help-seeking could have on military careers, similarly to reporting IPVA.

*If you talk about these things [mental health or suicide] in the military, they tend to look down on you because they think you are not as strong as you should be. Even though they say about all this help now, I still don’t think you get the help that they actually need.* (P5)

*I convinced him to go to [the military hospital] and get looked at, and obviously that is run by the military sergeants [0:30:08] the nurses. They just told him to man up. So it took me a long, long time to get him to go and see anybody ever again.* (P6)

##### Credibility

Participants identified that a fear of not being believed was a significant barrier to seeking help. Facilitators of help-seeking in some cases included having physical injuries, which participants felt added to their credibility. This was related to a general lack of awareness of the prevalence and impact of psychological abuse, which participants expressed was harder to prove and impacted them longer-term.

*I didn’t actually say anything until I turned up at work with a black eye. Then, after that, everything seemed a little bit easier […] because people could see, especially with it being the physical violence. […] But I do know the mental side of it is probably worse.* (P10)

*When it is coercive abuse, […] that can be far more damaging than physical […] ‘Oh yes, she’s in hospital, she’s got a fractured jaw. She’s definitely been abused.’ But, with my abuse, it was a lot harder to prove it, but it is far more devastating because it just affects your everyday, your mental health and your wellbeing. Everything.* (P9)

### Theme 3: Experiences of Services

Theme 3 describes participant experiences and perceptions of the pathways to formal sources of support. Four subthemes were derived: *Military health and welfare services; Civilian health and welfare services*; *Police and the Justice system;* and *Military/civilian divide*.

#### Military Health and Welfare Services

Participants reported seeking support via military-welfare charities, military health or welfare services, Chain of Command and other members of the military community, such as Chaplains. Most participants who sought support from military services felt that relationship difficulties among personnel and their families were not acknowledged. Participants repeatedly described a lack of protection for civilian victims, with perceptions that military services are tailored to and ‘protected’ for personnel only. This perception was heightened for partners of reservist personnel.

*There was nothing. When I rang up for [1:33:18] offer us support […] the words were, ‘You have to go to somebody within the civilians because you are a reservist wife, and we don’t do anything for reservists.’* (P7)

Most participants shared experiences of being discouraged from reporting IPVA or encouraged to stay in the relationship by military welfare staff, drawing on participant fears about potential consequences to their ex-partners career. Participants also recalled instances where welfare staff were dismissive, minimising the abuse they were disclosing and finding excuses relating to military training or military trauma for their (ex)partners’ behaviour.

*[When I tried to report it to military police] they sat there and made all the right noises. They kind of questioned me as well as to was I exaggerating, […] did I really want to press charges, did I really want to risk his career. […] They twisted things back that I was telling them: ‘No, but that just means he cares.’ So I did go back home confused, and, as I said, a couple of days later, he was told [that I had spoken to them].* (P23)

*I have had a families’ officer say to me, ‘I don’t know what you expect me to do. You’re living with a trained killer.’ […] ‘Well, he’s got PTSD, so that’s why it’s happened.’* (P8)

Participants described experiences of seeking help from military services in which they felt exposed, and potentially at greater risk, by the interviewer’s insensitive and unskilled questioning. They also described perceptions of collusion between the military agencies and personnel. In some cases, participants shared that although they had supportive interactions with welfare officers and the military police, there was no confidentiality and their (ex)partners were informed, resulting in them being more afraid to seek help in the future or leave. These experiences, which reflected a lack of understanding and awareness of IPVA, resulted in a lack of appropriate support, which facilitated controlling behaviour and contributed to participant mistrust of services.

*I managed to get him to go to marriage guidance. […] Through military, which maybe was a mistake. […] It was very formal. And my husband was sitting on my left-hand side, and this chap looked at me and said, ‘[…] you’re sounding like an abused wife. Has your husband ever hit you?’ He was sitting there. What am I going to say?* (P4)

*I would go to my families’ officer and disclose about the violence, and, before I have got home, I have already had an answerphone message from my husband telling me, ‘The welfare officer has contacted my sergeant. I know what you’re saying’ […] So it hugely puts people at risk because there is no confidentiality.* (P8)

Participants perceived that the military endeavoured to deal with personnel issues “*in-house*” in order to protect personnel, with only a minority reporting that their (ex)partners faced professional consequences for the IPVA perpetrated. Mostly participants reported minimal repercussions which might have encouraged behavioural change and described a context which instead facilitated abuse. Among the minority of participants describing sanctions against their ex-partner, these were perceived as being unfairly lenient, allowing for perpetuation of IPVA behaviours within relationships.

*I think that it got to the extreme because he was never reprimanded through the military. […] because he was in the military, he was given the excuses that he needed and the support that he needed in order to carry on.* (P8)

*There was no real punishment for him. […] it just gives people in the military even more excuse to behave in the way that they do and not change their ways because they know that there is not going to be any impact to their career whatsoever*. (P21)

#### Civilian Health and Welfare Services (Charity, Housing, Social and NHS Services)

Most participants described engaging with civilian charities and/or health professionals (NHS primary care system). Most felt satisfied once they received the support they required, both for psychological difficulties and for practical issues, such as safe housing and financial and legal advice.

*I got help with the normal everyday stuff: housing, finance, that sort of thing. But, more importantly, just help to rebuild who I am from inside again. […] The local council do what they call the* [name] *course, which they just go through all the things about how to spot, in potential partners, abusive behaviour*. (P12)

Nevertheless, some participants explained how they felt that many civilian health and welfare services were not fully equipped to support IPVA victim-survivors and highlighted gaps in services and expertise. Some described accessing support during periods of crisis but that this was short-term and there was no follow-up, which speaks to a lack of continuity of care.

*I have been round in circles for two or three years. […] Your GP just wants to give you pills. Most organisations have told me that my problems are too specific for them.* (P11)

*I think there is definitely a lack of ongoing support. I think, maybe when things are happening, whether it is the disclosure or just a marriage breakdown, I suppose you can get the support straightaway, but it doesn’t stay. It is not there long enough.* (P11)

Participants described how they had felt dismissed by services or that the staff were ill-equipped to manage or understand their relationship difficulties or mental health problems related to their experiences of IPVA, and identified a lack of signposting to specialist IPVA services. This was also described in relation to gaps in safeguarding procedures and a lack of onward referrals.

*I think it was during the pregnancy, I spoke to the midwife and I spoke to the GP. I think maybe only once, though, because I was quite worried. […] I think that is when everything started to change, really. I did feel quite controlled and trapped. […] I think I got some time off work. I think that was it. […]they signed me off for pregnancy-related illness* (P24)

*We did go to [relationship counselling service], but, as soon as they found out about his behaviour, they refused to see us anymore. […] Instead of saying, ‘This is domestic abuse, we’re contacting the police on your behalf,’ or, ‘We’re contacting social services because we have concerns about your children’s safety,’ they just put their hands up and said, ‘Sorry, we don’t deal with domestic abusers. We won’t see you anymore.’* (P20)

Some who accessed mental health support reported it to be beneficial once in the system but described significant delays in accessing appropriate support. Others shared that long waiting lists for mental health support through the NHS resulted in a need to seek help privately, and was thus only possible for the short term due to financial constraints.

*After about four months is when [the mental health authority] finally contacted me. Then I was put on a waiting list for almost a year, and then they called me in to do the initial assessment.* (P18)

*I paid for private counselling […] Only three because I couldn’t afford it, to be honest, and through the GP was such a long wait*. (P2)

Participants described how a lack of continuity in care in the NHS was not conducive to building the trusting relationships with clinicians required to encourage disclosure of relationship abuse. Only a minority of participants described disclosing the abuse when seeking medical attention, with most claiming other causes for their injuries.

*The NHS is hopeless in that way because you don’t ever get to see the same person more than once, so you don’t get to build a relationship with them. So, no, not really. *(P17)

*I have actually been to hospital as a result of the abuse, but I have lied about what happened*. (P4)

Some participants reported that support for their children for psychological and behavioural difficulties (e.g., mood or sleep disturbances) resulting from witnessing or experiencing abuse at home was easier to access than support for themselves. They felt that services to support children are better structured and they felt that the pathway to care was well established.

*I have managed to get the support for my children because that is a little bit easier to access, but not so much for me, no.* (P11)

Participants described experiences of overcoming multiple barriers to accessing support, including limited service capacity and navigating clinical thresholds. The minority of participants who described wanting to save their relationship additionally highlighted limited opportunities for interventions beyond encouraging participants to leave.

* [A Domestic Abuse charity] had wanted me to leave him long before that, and, because I wouldn’t leave, they wouldn’t help. […] It has been really hard because […] once I wasn’t a high-risk person, after I had left him, […] they kind of withdrew all help. I have had to phone and email them repeatedly, and I still haven’t had the help I needed.* (P24)

*It was very much a case of, if you choose to stay, then you are on your own, really.* (P8)

#### Police and The Justice System

For the majority of participants, the police were called as a result of the IPVA. Some participants described positive experiences of being cared for by police.

*The lady at the police station was brilliant. I don’t know if she was a PC or a sergeant, but I know she was brilliant, and believed me, which was amazing.* (P10)

Others described a lack of police follow-up and expressed that they felt stigmatised in their encounters with police or that their experiences were dismissed when they were actively encouraged to return to the relationship.

*The police were terrible. I actually had a policeman say, ‘Look, we’re here with your husband and your daughter, your husband just says come home.’* (P12)

*When the policeman said to me, ‘Gosh, you’re not the normal type of domestic violence. You’re both professional working people,’ I felt that there was stigma attached to DV; it only happens to people on council estates, and that is so not true.* (P2)

Once in the legal system, participants described a perceived lack of IPVA awareness and victim support within the justice system. A minority of participants discussed being cross-examined by their (ex)partners in court, which contributed to a perceived lack of victim protection. Others reported how the presence of military support in court may have mitigated against more severe punishment for offences related to IPVA within the civilian system due to perceptions of personnel as heroes or victims.

*Your perpetrator can drag you back to the court numerous times on some charge, and you have to go, and you have to stand there with him, so he carries on abusing you. So there was no protection for me, and it hasn’t been to other victims either. (*P9)

*Military men don’t go to prison for domestic violence, because the military will address the court and say he is a changed man, he is a very good soldier, he has got lots to contribute, he has deployed to all these places, he is basically Queen and country and all the rest of it. And judges are very influenced by that […] He is still serving*. (P8)

#### Military/Civilian Divide

A few participants noted that encounters with police highlighted confusion within civilian responders about the boundaries between civilian and military law, which created gaps between services. Participants also shared that public perceptions of the impact of personnel experiences in combat operations elicited expressions of sympathy towards their (ex)partners.

[The police] *recommendations were just, ‘Speak to the army. The army will sort it out.’ That was basically their recommendation. Their stock answer to everything was, ‘Well, he’s been to Afghanistan. I can see why he’s angry all the time.’* (P19)

Participants described attempts by the military to shield personnel, using their authority to dissuade civilian police from prosecuting or “*closing ranks*”. The military was perceived by participants as wanting to protect their employees and avoid negative publicity rather than address IPVA and its consequences.

*They [the military] supported him. They obviously went to court with him. He kept his job. […] he did actually go abroad when the police were looking for him […] They actually had to get Interpol involved to get him back because the military tried to keep him out of the country so the police couldn’t talk to him.* (P2)

*I think the military looks after its own, is the bottom line, and the military very much, especially in recent years, wants to portray to the British public that they are this amazing organisation, and so they just want to brush anything negative under the carpet.* (P8)

A symptom of the military/civilian divide and barrier to prosecution described by participants was both the closed nature of military records of offences or domestic incidents committed by military personnel and the separate military and civilian court systems. This was perceived to contribute to (ex)partners not experiencing the same repercussions as they might do without military protection.

*I think they [Military] have got to stop brushing it under the carpet. […] There is no record anywhere of […] the Royal Military Police coming round and dragging him off. This isn’t on record anywhere, not for anybody to get access. It can’t be used. It is hidden away*. (P12)

## Discussion

This study explored experiences of help-seeking for IPVA among the civilian partners of military serving or ex-serving personnel. Three main themes were identified, describing the drivers and barriers to help-seeking and experiences of services. The findings demonstrate that civilian victim-survivors of IPVA by military personnel report many of the help-seeking experiences documented in the civilian victim-survivor literature, but also stress the additional difficulties experienced as a result of being in a relationship with military personnel and on the boundaries of civilian and military communities (Table 2).

Many of the motivations to help-seeking in our sample depicted in Theme 1 ‘*Drivers of help-seeking*' echoed findings from research with civilian victim-survivors who were not in relationships with military personnel and included an escalation in the nature and frequency of the abuse and recognition of the impact of abuse on children (Evans & Feder, 2016). As in civilian research, some described friends and family to be instrumental in supporting participants to initiate contact with services (Ansara & Hindin, 2010). Many of the barriers to help-seeking for IPVA (see Theme 2 ‘Barriers to help-seeking') were similarly shared with civilian IPVA help-seeking research, including: lack of understanding of IPVA, particularly of psychological abuse and gas-lighting (Sweet, 2019); hope that the abuse would end and guilt over breaking up the family unit (Dare et al., 2013; Eckstein, 2011); stigma, shame and fear of not being believed (Murray et al., 2018; Overstreet & Quinn, 2013; Rose et al., 2011); and lack of confidence in services and mistrust related to experiences of victim-blaming (Huntley et al., 2019; Meyer, 2016).

Beyond the barriers to help-seeking which appeared to be common to victim-survivors in military and non-military relationships, the participants’ narratives revealed the impact of military specific factors on their experiences of help-seeking which complicate their IPVA help-seeking journey and lead to experiences more unique to this cohort. The participants reflected on the influence that the wider military community had on expectations of them as partners of military personnel, which are likely to have reinforced some of the psychological barriers to leaving abusive relationships. For example, prioritising the needs of their military partners over their own and protecting the military family unit (Alves-Costa et al., 2021) and cultural ideals of loyalty (Kern, 2017) may keep spouses in abusive military relationships. The normalisation and minimisation of violence and aggression in the military community, as described by civilian victim-survivors of IPVA perpetrated by serving or ex-serving military personnel, is also reported to extend to the abuse within relationships (Alves-Costa et al., 2021) and is likely to have amplified the barriers described, such as lack of understanding and recognition of non-physical abuse within relationships, delayed help-seeking, and contributed to the participants’ tolerance of objectively moderate to severe IPVA experiences before seeking help. Some barriers which overlap with those identified in the civilian literature, such as fears that their own credibility would be questioned (Murray et al., 2018; Overstreet & Quinn, 2013; Rose et al., 2011), were compounded by the perception that the public sympathise with military personnel and that military services prioritise personnel over families and partners. Furthermore, in addition to the significant impact of IPVA on psychological functioning and poor mental health and its role in the occurrence and exacerbation of IPVA described by this sample (Alves-Costa et al., 2021), participants highlighted that barriers and delays to help-seeking for mental ill-health contributed to perpetuate their experiences of abuse and delay help-seeking for IPVA. Findings suggested that societal stigma associated with help-seeking for both IPVA and mental health difficulties is magnified in hypermasculine military environments, as has been described in other stereotypically masculine occupational settings (e.g. law enforcement: White et al. (2016); first responders: Haugen et al. (2017).

Other barriers to help-seeking experienced by participants seemed more specific to their (ex)partner’s military service. As described in research with civilian partners in abusive relationships with military personnel by Williamson (2012), most participants in this study noted that they feared the impact on their (ex)partners’ careers in addition to fear of their (ex)partner, and hence did not seek support. Participants identified that dependency on their (ex)partner impaired their ability to seek help and perpetuated the cycle of IPVA. Financial dependency and isolation may be a particular barrier for the civilian partners of military personnel, as frequent military-related relocations have been found to disrupt spouses’ social networks and ability to maintain employment (Blakely et al., 2014; Gribble et al., 2019) and the military may provide housing and other welfare support (Sparrow et al., 2020), which they would lose if the relationship ended. Adding further weight to previous study findings of help-seeking for IPVA in the military community, these barriers were especially heightened for FCO participants, whose circumstances, such as a reliance on their (ex)partner both financially and for the right to remain in the country, as well as their isolation from their own communities, impaired their ability to seek help (Gray, 2016; Sparrow et al., 2020). These factors may be of heightened significance in the context of the Covid-19 pandemic due to local lockdown rules and social distancing, international border closures or travel limitations and the potential for financial instability and job loss.

Many participants reported accessing NHS services and third sector civilian and military charities for support, with a minority also seeking help from military welfare services (see Theme 3 ‘Experiences of help-seeking'). Experiences of accessing support were mixed and participant narratives echoed those of victim-survivors outside of the military community, reemphasizing the wider difficulties in identifying and managing IPVA highlighted in the civilian literature. For instance, participants observed that their consultations with services were not conducive to self-disclosure of abuse, a lack of signposting and onwards referrals when disclosure did take place, and gaps in service provision and delays, resulting from long waitlists or not meeting clinical thresholds, which impaired their access to support (Trevillion et al., 2012; Williams et al., 2017). Some participants described perceptions of victim stigmatisation and a lack of victim protection, not being taken seriously or believed, being blamed for having stayed in the abusive relationship, or perceiving that the violence was normalised, minimised or excused. Some of these experiences are also common with victim-survivors more broadly (Huntley et al., 2019; Meyer, 2016). Participants also identified a lack of support for those attempting to resolve their relationship difficulties and remain with their partners, marking this group as particularly vulnerable to not receiving appropriate interventions. These problems were related to a wider lack of awareness and understanding of IPVA within services as has been extensively reported in civilian populations (Keeling & Fisher, 2015; Ramachandran et al., 2013; Rose et al., 2011; Sparrow et al., 2020; Sprague et al., 2012).

In line with findings from wider IPVA research in military communities (e.g. Gray, 2016; Kern, 2017; Lane et al., under review-a; Sparrow et al., 2020; Williamson & Matolcsi, 2019), most participants who sought support from military services described feeling let down and that attitudes to IPVA within the military community were a barrier to help-seeking, calling for culture change in organisational-level attitudes towards civilian spouses and partners. Participants described difficulties accessing military services as civilians and this was particularly noted by those in relationships with reservists, a problem of “*falling between the cracks*”, which has been documented in wider reservist family research (Cunningham-Burley et al., 2018). Furthermore, perceived lack of confidentiality and collusion between military services and participants’ military partners was reported, supporting previous research which also observed that “*safe places”* are not always perceived as *“safe*” (Gray, 2015, 2016; Kern, 2017; Williamson, 2012; Williamson & Matolcsi, 2019). There was a perception that military welfare services prioritised personnel and the maintenance of the family unit, in some cases excusing IPVA and discouraging participants from reporting their (ex)partners despite the risks this brings to victim-survivors, also described by Gray (2015) in the UK and Kern (2017) in the US, perpetuating the cycle of self-blame and resistance to help seeking. This also extended to support and representation from the military in criminal justice settings, which was regarded by participants to contribute to (ex)partners receiving relatively light punishments. These difficulties were perceived to be amplified by the gaps between civilian and military law, with many participants describing difficulties with help-seeking pertaining to a perceived military/civilian divide, conceptualised in research by Gray (2016) and Rahbek-Clemmensen et al. (2012). For example, participants described being redirected between civilian and military police with no apparent communication of information between the two services, and being unable to access closed military records, which impacted on the timescale and success of prosecution. This disconnect between civilian and military services was perceived by participants to enable the military to “*close ranks*” and protect personnel, favouring preserving a positive public image and managing IPVA “*in house*”, as described previously by military health and welfare staff (Sparrow et al., 2020).

### Strengths and Limitations

The research provides further understanding of participant perceptions of the influence of the military context on their experiences of help-seeking. PPI involvement supported the development of the interview guide and the validation of the findings, minimising risk of researcher bias. Nevertheless, limitations of the research include the homogenous sample of predominantly White women in heterosexual relationships with male serving personnel or veterans, almost all reporting unidirectional moderate to severe abuse. In drawing interpretations and making recommendations, we must therefore acknowledge the restricted range of narratives on which our findings are based. For example, research has identified differences in help-seeking approaches for IPVA and service use according to ethnicity (Flicker et al., 2011). Our findings stress that increased awareness and understanding of IPVA is needed within civilian and military services providing health and welfare support, as well as within military communities themselves, supporting priority areas of the military Domestic Abuse Strategy (MOD, 2018).

Further mixed-methods research on military relationships and IPVA using larger, more varied samples is needed to investigate the help-seeking experiences of male victim-survivors, LGBT + couples, victim-survivors from minority ethnic groups, as well as those of military personnel victim-survivors of IPVA.

### Implications and Recommendations

This study represents one of the first UK qualitative research studies exploring civilian experiences of help-seeking for IPVA and its related difficulties following IPVA perpetrated by a serving or ex-serving military (ex)partner. The research provides further understanding of participant perceptions of the influence of the military context on their experiences of IPVA, help-seeking and identifies implications for policy, practice and future research.

The UK Domestic Abuse Act (Home Office, 2021) seeks to address many of the challenges in providing support to victim-survivors of IPVA. The military has in recent years recognised their responsibility to provide better support to military families, acknowledging the imperative to tackle IPVA within its community (MOD, 2018). The new Domestic Abuse Strategy provides an opportunity for the military to examine how military specific factors affect intimate relationships and the risk of IPVA, and to consider how to reduce barriers to help-seeking and improve experiences of support services for civilian partners of military personnel. Our findings reinforce the need for a military specific strategy to tackle IPVA by providing insight into the additional or heightened challenges civilian partners in abusive relationships with military personnel may face when seeking help for IPVA. The Ministry of Defence are implementing their first Domestic Abuse strategy (Ministry of Defence, 2018) and studies such as this will contribute to a stronger evidence base on which to base forthcoming reviews. Our findings stress that increased awareness and understanding of IPVA is needed within civilian and military services providing health and welfare support, as well as within the military communities themselves. Lack of understanding of IPVA was identified not only a barrier to help-seeking but was also a factor negatively impacting civilian partners’ experience of service response to disclosure (see Theme 2, subthemes 1 ‘Individual-level barriers’ and 4 ‘Societal barriers’ and Theme 3 ‘Experiences of services’). Education should be available to personnel and military families as part of training/well-being packages, for instance on HIVEs in military bases, especially in anticipation of key risk periods, such as the peri-deployment period and transition out of service (Alves-Costa et al., 2021). We endorse the recent uplift in training in IPVA within some military health and welfare services and recommend that this is even more widely available across the UK irrespective of geographical location. Particular attention should be paid to training in the identification and management of non-physical IPVA and the wider impact of IPVA on the mental health of victim-survivors and children. Increased understanding can help to reduce victim-blaming and the risk of re-traumatisation by services, as well as eliminate barriers to help-seeking, as described by our participants. Removing any stigma and real or perceived barriers to reporting domestic abuse is currently a long-term plan highlighted in the military Domestic Abuse Strategy (2018) and is being driven through policy updates and awareness campaigns. Mandatory IPVA training for all staff, with additional training for line managers and health and welfare professionals, would support these efforts. Our findings stress that increased awareness and understanding of IPVA is needed within civilian and military services providing health and welfare support, as well as within military communities themselves, supporting priority areas of the military Domestic Abuse Strategy (MOD, 2018).

Participants highlighted their difficulties trying to identify, access and navigate multiple services for individual IPVA-related difficulties (see Theme 2, subtheme 3 ‘Service-related barriers’ and Theme 3 ‘Experiences of services’), raising the need for parity of access to IPVA services for civilian partners of military personnel, including those of reserve personnel, with clearly delineated pathways to support. The latter calls for a more co-ordinated response by support services. First line health and welfare staff, both military and civilian, with the skills to screen for and identify IPVA, and signpost to appropriate specialist services where necessary are crucial to this response, in addition to cross-agency working across military, civilian and third sector health, welfare and DVA support agencies. Given the significance of mental-health difficulties, both in their contribution to the occurrence, exacerbation or perpetuation of IPVA and as a consequence, it is crucial that mental health professionals are alert to IPVA in their patients’ histories or current presentations and have the confidence and skills to enquire about it in their routine clinical interactions (Hegarty et al., 2020). Both military and civilian services must be more openly and widely advertised. A national IPVA awareness campaign within the military could provide a helpful impetus for improved awareness and would complement efforts made to date on military Family Federation websites. As highlighted by the MOD Domestic Abuse Strategy (2018), collaboration with civilian services is recommended to improve understanding within these services of the unique aspects of military life and provide tailored, person-centred support. Certain couples were highlighted in our findings as needing extra support or not currently receiving appropriate support. Particular consideration is needed for the support given to FCO civilian partners and families, who may be more vulnerable to IPVA and lacking in resources to resolve their situations as a result of increased financial dependency on military personnel and social isolation, as demonstrated by our findings (see Theme 2 ‘Barriers to help-seeking'). Victim-survivors of IPVA who want to remain with their partners was highlighted as another under-supported group. We know from civilian research that this is not an uncommon problem (Sparrow et al., 2020) and needs to be considered as a priority given emerging evidence in support of working with some couples to improve relationship functioning (Taft et al., 2016).

Furthermore, independent and confidential support for partners and families, regardless of relationship and civilian status, may help to reduce barriers to accessing both mental health and relationship support arising from stigma and fear of potential impact on their partners’ military careers (see Theme 2, subthemes 1 *‘*Individual-level barriers’ and 2 ‘Relationship barriers’, and Theme 3, subtheme 1 ‘Military health and welfare services’). More accessible support, which is confidential from the chain of command, is required. This does not appear as part of the MOD Domestic Abuse Strategy (2018), and we would urge services to consider such provision to facilitate disclosure and help-seeking. Also, the use of Domestic Abuse Advocates, independent of the military, who have specialist skills in the assessment and management of IPVA is a strategy not yet implemented by the UK military as in civilian settings (Feder et al., 2011; Malpass et al., 2014), but has been recommended by UK military health and welfare workers in previous research by this group (Sparrow et al., 2020). Criticisms of both the military and the civilian justice systems also need to be addressed (see Theme 3, subthemes 3 ‘Police and the criminal justice system’ and 4 ‘Military / civilian divide’). Concerns that IPVA by military personnel is not always appropriately investigated or sanctioned within the military judicial system and that within the civilian justice system military service may be used inappropriately in mitigation highlight the need for an investigation of the handling of IPVA cases across both jurisdictions to ensure transparency, fairness and consistency. A better understanding of the influence (or not) of military service in each individual case of IPVA is essential to inform just sentencing. The wide-reaching impacts of the bureaucratic divide between the military and civilian justice systems need to be examined. For example, restricted access to military records may inhibit the implementation of Domestic Violence Disclosure Scheme (Home Office, 2020), which enables the police to disclose information to a victim or potential victim of domestic abuse about their partner’s or ex-partner’s previous abusive or violent offending.

Lastly, research has a vital role in informing and evaluating changes to IPVA support services for this population, driving innovative evidence-based practice and ensuring good outcomes are established and maintained. Further mixed-methods research considering experiences of civilian male victim-survivors, LGBT + couples and victim-survivors from minority ethnic groups is needed to explore their help-seeking journeys for IPVA in the context of military relationships to ensure that care provision is tailored to their needs and to continue to inform the Government Ministry of Defence Domestic Abuse policy.

## Conclusion

These results highlight the challenges faced by civilian victim-survivors when seeking help for IPVA and how being in an abusive relationship with someone in the military can magnify some of those challenges and give rise to different experiences of help-seeking. Participants experiences suggest that a shift in attitude to and understanding of IPVA is needed from the top down in the military and action taken to reduce barriers to help-seeking by civilian partners, improve access to and experience of support services and ensure that due legal process is facilitated. The new Ministry of Defence Domestic Abuse Strategy is evidence of the motivation to make such changes and to provide support for military families including for victim-survivors, perpetrators and children. The recommendations which arise from this study should inform further review of that strategy.
